# Fossilized spermatozoa preserved in a 50-Myr-old annelid cocoon from Antarctica

**DOI:** 10.1098/rsbl.2015.0431

**Published:** 2015-07

**Authors:** Benjamin Bomfleur, Thomas Mörs, Marco Ferraguti, Marcelo A. Reguero, Stephen McLoughlin

**Affiliations:** 1 Department of Palaeobiology, Swedish Museum of Natural History, Stockholm, Sweden; 2 Dipartimento di Bioscienze, Università degli Studi di Milano, Milano, Italy; 3 División Paleontología de Vertebrados, Museo de La Plata, La Plata, Argentina; 4 CONICET: Consejo Nacional de Investigaciones Científicas y Técnicas, Argentina; 5 Instituto Antártico Argentino, Balcarce 290, (C1064AAF), Buenos Aires, Argentina

**Keywords:** Annelida, Clitellata, fossilization, spermatozoa, taphonomy, Antarctica

## Abstract

The origin and evolution of clitellate annelids—earthworms, leeches and their relatives—is poorly understood, partly because body fossils of these delicate organisms are exceedingly rare. The distinctive egg cases (cocoons) of Clitellata, however, are relatively common in the fossil record, although their potential for phylogenetic studies has remained largely unexplored. Here, we report the remarkable discovery of fossilized spermatozoa preserved within the secreted wall layers of a 50-Myr-old clitellate cocoon from Antarctica, representing the oldest fossil animal sperm yet known. Sperm characters are highly informative for the classification of extant Annelida. The Antarctic fossil spermatozoa have several features that point to affinities with the peculiar, leech-like ‘crayfish worms' (Branchiobdellida). We anticipate that systematic surveys of cocoon fossils coupled with advances in non-destructive analytical methods may open a new window into the evolution of minute, soft-bodied life forms that are otherwise only rarely observed in the fossil record.

## 1. Background

Despite recent advances in molecular phylogenetics [1–4], the evolutionary history of Clitellata—earthworms, leeches and their relatives—is still poorly understood. This is in part because the delicate bodies of clitellates consist almost entirely of soft tissues, and can thus become fossilized only under exceptional circumstances [5]. Nevertheless, these organisms have left a peculiar presence in the fossil record in the form of dispersed egg cases (cocoons), which are very resistant to physical and chemical decay [6]. Clitellate cocoons are common, though sporadically illustrated, components of plant micro- and mesofossil assemblages obtained via bulk dissolution of clastic sedimentary rocks [6–9] as old as Middle Triassic [10]. This potential source of information about the origin and evolutionary history of clitellates has, however, received little scientific attention thus far, because it would appear impossible to determine the systematic affinities of the cocoon producers in any greater detail based on morphology alone.

Here, we report the occurrence of fossil spermatozoa that are preserved embedded in the wall layers of a 50-Myr-old (early Eocene) clitellate cocoon from Antarctica. These are the oldest fossil animal spermatozoa yet identified. Some morphological features of the spermatozoa are reminiscent of those of extant Branchiobdellida, a peculiar group of leech-like worms whose extant representatives are ectosymbionts on freshwater crayfish.

## 2. Material and methods

The fossil Clitellata cocoons were collected from marginal-marine deposits of the La Meseta Formation, Seymour/Marambio Island, Antarctic Peninsula (e.g. [11]; electronic supplementary material, figure S1). Individual cocoons were picked from dry-sieved sediment samples of poorly consolidated, shelly conglomerate informally referred to as the ‘*Natica* horizon’. The age of this deposit is considered to be approximately 50 Ma (Ypresian, early Eocene) based on strontium isotope dating and mammal biostratigraphy (e.g. [11]; see the electronic supplementary material). The fossils were analysed via light and scanning electron microscopy and via synchrotron-radiation-based X-ray tomographic microscopy (electronic supplementary material).

## 3. Results

**Figure 1.**
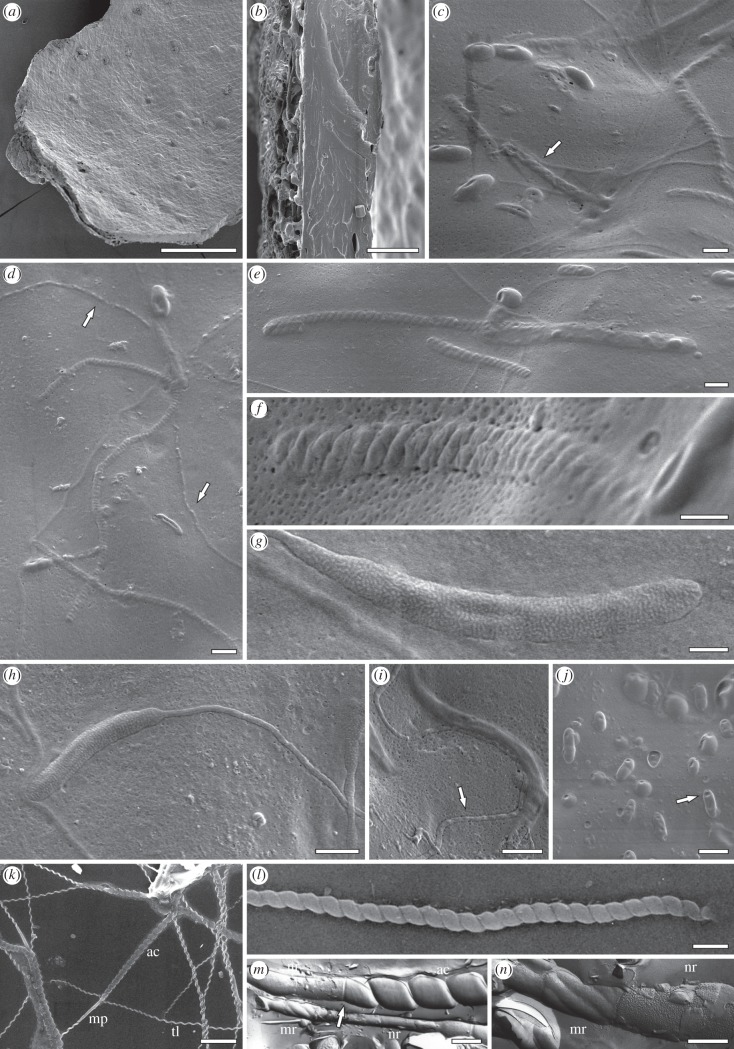
Scanning electron micrographs of the Antarctic annelid-cocoon fossils showing cocoon structure and included spermatozoan fragments and bacteria (*a–j*), with images of extant branchiobdellid spermatozoa for comparison (*k–n*). (*a*) Overview of cocoon fragment. (*b*) Fracture surface showing spongy outer (left) and compact inner (right) wall layers. (*c*) Encased bacteria and spermatozoan fragments; note element showing multiple coils (arrow). (*d*) Encased spermatozoan fragments with tail portions (arrows). (*e*) Encased spermatozoan fragments resembling acrosomes. (*f*) Encased spermatozoan fragment tangentially encased in the cocoon wall. (*g*) Spermatozoan fragment showing granular texture. (*h*) Spermatozoan fragment showing granular texture and attached tail portion. (*i*) Isolated tail portion showing beaded structure (arrow). (*j*) Encased rod-shaped bacilli with characteristic dimples (arrow). (*k*–*n*) Spermatozoa of *Branchiobdella* sp. showing ‘drill-bit’ type acrosomes (ac), mid-pieces (mp) with nuclear (nr) and mitochondrial regions (mr), and tail regions (tl); note the suture between acrosome and nuclear region in (*m*) (arrow). Scale bars: (*a*) = 250 µm; (*b*) = 20 µm; (*c*–*e*,*h*,*i*,*m*) = 1 µm; (*f*,*g*,*n*) = 500 nm; (*j*,*l*) = 2 µm; (*k*) = 10 µm.

A single, approximately 1.5 × 0.8 mm, small annelid-cocoon fragment (specimen NRM-S089729) exposes the inner surface of the cocoon wall (figure 1*a*). The wall consists of a more than 25-µm-thick solid inner layer enveloped by a 5–10-µm-thick spongy outer layer composed of a loosely amalgamated network of interwoven ‘cables' up to approximately 5 µm in diameter [12] (figure 1*b*).

SEM analysis revealed various micro- and nano-inclusions embedded in the inner layer of the cocoon wall (figure 1*c*). The most conspicuous biological inclusions are fragments of straight or variably bent, narrowly cylindrical elements that reach approximately 18 µm long by approximately 600 nm wide and have a characteristic, helical ‘drill-bit’ structure, in some cases containing more than 80 gyres each with indistinct transverse striations (figure 1*d*–*f*). Other elements are rod-shaped, up to 12 µm long and approximately 500 nm wide, and with a finely granular texture (figure 1*g*,*h*). These commonly bear a whip-like tail up to approximately 250 nm thick and more than 30 µm long (figure 1*h*). Where these tails are well preserved and sharply defined, they reveal a regular beaded morphology reminiscent of a helical anatomical structure (figure 1*i*). In addition, we found a few approximately 5-µm-long and approximately 400-nm-wide, isolated elements that are composed of several intertwined, coiled fibres (figure 1*c*) that have a significantly lesser angle of coiling than the rather strongly compressed, simple spiral of the ‘drill-bit’ elements (figure 1*d*–*f*). Associated fossil bacteria consist mostly of rod-shaped, approximately 2-µm-long and approximately 0.8-µm-wide bacilli (figure 1*j*); many bear a characteristic dimple on the surface (figure 1*j*, arrow), and some occur in chains or clusters. Additionally, we attempted synchrotron-radiation-based X-ray tomographic microscopy on a portion of a cocoon wall; possible spermatozoa were detectable, but only at the limit of the instrument's resolution (see electronic supplementary material, figure S2).

## 4. Discussion

Similarities in dimensions, structure and texture indicate that the isolated elements described above represent the various components of the specialized filiform spermatozoa typical of clitellate annelids. Being very short-lived and delicate structures, spermatozoa are very rare in the fossil record. Perhaps the best-documented examples are spermatozoids of early land plants from the Devonian Rhynie Chert [13] and of gymnosperms from the Permian of Australia [14]. Preservation of animal spermatozoa is exceedingly rare, with just single pre-Quaternary records of collembolan spermatozoa from late Eocene Baltic amber [15] and of phosphatized giant spermatozoa of ostracods from Miocene cave deposits of Australia [16]. The clitellate spermatozoa described herein thus constitute the oldest fossil animal spermatozoa yet recorded, predating the previous oldest occurrence (late Eocene [15]) by at least 10 Myr.

Interestingly, of all morphological features that the annelid body plan exhibits, those that are currently considered among the most informative for resolving systematic relationships relate to the morphology and ultrastructure of the spermatozoa [2,17–20]. However, detailed comparisons of the fossils with extant taxa are difficult at present for several reasons: first, although progressively more living taxa are being sampled [21], our knowledge of the structural diversity of extant clitellate spermatozoa is still very incomplete; second, the fossil material consists mostly of disarticulated remains that yield limited information about the architecture of the complete spermatozoa; and third, the study of extant material has relied largely on TEM analysis to resolve ultrastructural features, whereas little is known about the gross morphology and texture of spermatozoa when studied using SEM, as is the case with the fossil material. We surmise that future detailed analyses of fossil cocoon inclusions, perhaps using nanotomographic methods (e.g. [16]), might reveal preservation of ultrastructural features in embedded spermatozoa, which would provide a great leap forward for precisely identifying cocoon-producing annelids in the fossil record.

Despite these constraints, we contend that several features of the fossil spermatozoa point to probable affinities of the cocoon producer with the peculiar ‘crayfish worms' (Branchiobdellida). The long, conspicuous ‘drill-bit’ structures (figure 1*d–f*) are very similar to the characteristic, greatly elongate acrosomes of *Branchiobdella* (figure 1*k–m*; [20,22]), which in extreme cases can reach 90 µm in length, forming the longest acrosomes in nature [18]. Moreover, the beaded appearance of some flagella (figure 1*d*,*i*) may be due to the presence of a helical marginal fibre (figure 1*k*), a feature unique to the tails of branchiobdellid spermatozoa [19,20]. Finally, the granular surface texture of some elements anterior to the tail (figure 1*g*,*h*) matches that of the nuclear regions of branchiobdellid sperm as seen in freeze-dried material studied via SEM (figure 1*m*,*n*). Extant branchiobdellids are obligate symbionts of freshwater crayfish of the Northern Hemisphere, and their evolutionary and phylogeographic history is supposed to be closely linked to that of their host taxa [22]. Hence, their possible fossil occurrence in Antarctic freshwater ecoystems of the early Eocene greenhouse world would markedly extend their palaeobiogeographic distribution, indicating that their evolutionary history may be more complex than currently recognized.

**Figure 2.**
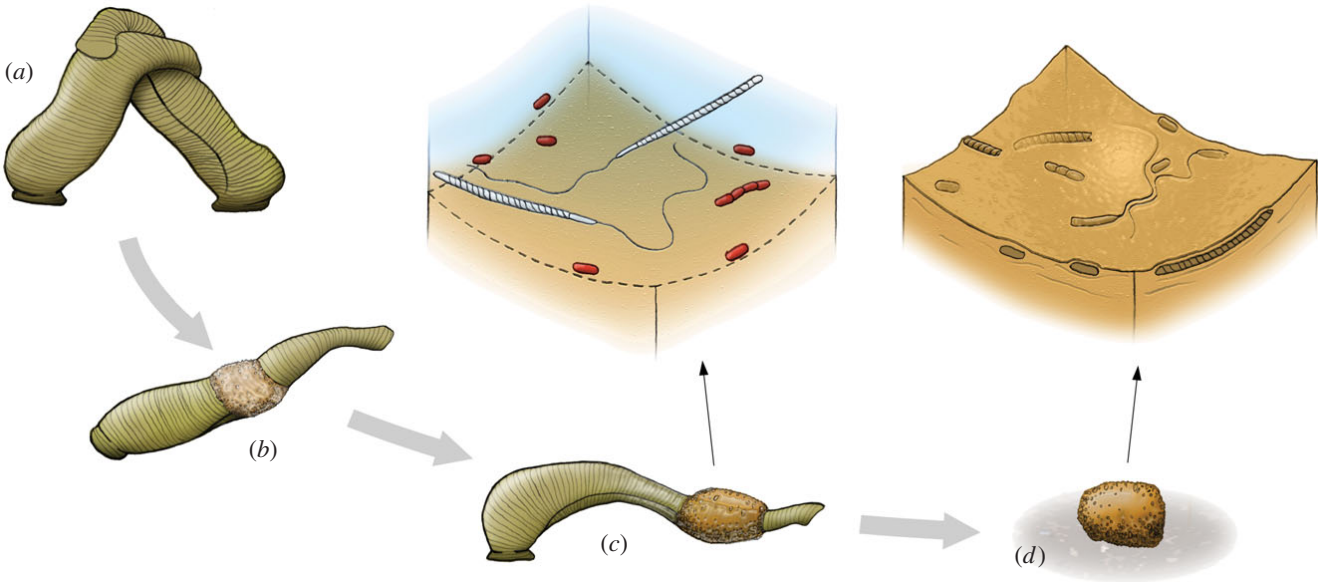
Diagram illustrating the inferred mode of fossilization of microorganisms in clitellate cocoons, exemplified by a common medicinal leech (reproductive stages modified from Sims [23]). (*a*) Two leeches mate; (*b*) a cocoon is secreted from the clitellum; (*c*) eggs and sperm are released into the cocoon before the animal retracts and eventually deposits the sealed cocoon on a suitable substrate (*d*). Insets depict enlargements of the inner cocoon-wall surface showing how spermatozoa and microbes become encased in the solidifying inner cocoon wall.

Clitellate annelids secrete their cocoons from the clitellum—a series of specialized body segments—initially in the form of a mucous substance onto which proteinaceous material is deposited in successive layers and in characteristic arrangements (figure 2; [12]). In most cases, eggs and sperm are then released into the cocoon by the hermaphroditic adult as the animal withdraws (figure 2*c*). The cocoon is finally sealed and deposited, and then cures over several hours to days to form a resistant egg case for the developing embryos [12]. Spermatozoa appear to commonly become entrapped together with other microorganisms inside the proteinaceous cocoon wall before the material is completely solidified (figure 2*c*,*d*). Two previous studies have identified microscopic soft-bodied organisms encased in the walls of fossil clitellate cocoons: a nematode in an Early Cretaceous cocoon from Svalbard [24] and a *Vorticella*-like ciliate protozoan in a Triassic cocoon from Antarctica [9]. This unusual fossilization process appears to be analogous to entombment in amber—both processes permitting three-dimensional preservation of hard- or soft-bodied microorganisms with very fine morphological and structural details [25]. Thus, clitellate annelid cocoons offer an outstanding and little-studied preservational medium for a potentially broad range of microscopic soft-bodied organisms of various freshwater, soil and leaf-litter habitats. We anticipate that systematic surveys of ancient cocoons may open a unique window into the evolutionary history of a range of soft-bodied microorganisms that otherwise lack a fossil record.

## Supplementary Material

Bomfleur_et_al_ESM
