# Supplementary material for: Fossilized spermatozoa preserved in a 50-Myr-old annelid cocoon from Antarctica
Source: Biol Lett. 2015 Jul;11(7):20150431. doi: 10.1098/rsbl.2015.0431 (PMC4528455; doi:10.1098/rsbl.2015.0431)
Supplement: Bomfleur_et_al_ESM [file rsbl20150431supp1.pdf]

Electronic supplementary material to

Fossilized spermatozoa preserved in a 50-my-old annelid cocoon from Antarctica

Benjamin Bomfleur[1]*, Thomas Mörs[1], Marco Ferraguti[2], Marcelo A. Reguero[3, 4, 5], and Stephen McLoughlin[1]

[1]Department of Palaeobiology, Swedish Museum of Natural History, Stockholm, Sweden; [2]Dipartimento di Bioscienze, Università degli Studi di Milano, Milano, Italy; [3]División Paleontología de Vertebrados, Museo de La Plata, La Plata, Argentina; [4]CONICET; [5]Instituto Antártico Argentino, Balcarce 290, (C1064AAF), Buenos Aires, Argentina

*****_author for correspondence; email: benjamin.bomfleur@nrm.se_

Geological and palaeontological background information

The La Meseta Formation (Seymour Island Group; Seymour/Marambio Island, Weddell Sea, Antarctic Peninsula; figure S1) consists of shallow-marine to estuarine, poorly consolidated siliciclastic deposits that represent the filling of an incised-valley system [26,27]. It contains rich and very diverse fossil assemblages dominated by marine invertebrates and vertebrates (see e.g. [12,26,27]. The fossil annelid cocoons were collected from localities IAA 1/90 (also known as the 'Ungulate site'; 64°14'04.67"S; 56°39'56.38"W) and IAA 2/95 (known as the 'Marsupial site'; 64°13'58"S; 56°39'06"W) from the northern part of the island; both sites expose lenses of a distinctive conglomerate with abundant shells of naticid gastropods ('*Natica* horizon'), and occur at the same stratigraphic level in the central portion of the Cucullaea I Allomember of the La Meseta Formation (figure S1). In addition to abundant marine fossils, the '*Natica* horizon' in particular has yielded a broad range of fossils of non-marine organisms, including remains of terrestrial mammals and plants [26,27]. Of special interest is the high abundance of dispersed seeds of aquatic angiosperms, such as *Nuphar* ('water lilies') and *Nelumbo* ('lotus') [28], which documents the existence of permanent freshwater bodies as suitable potential habitats for the cocoon-producing clitellates described here. Overall, palaeoclimatic reconstructions indicate temperate and generally ice-free conditions for the Antarctic Peninsula Region during the early Eocene [29].

Although the precise age of the La Meseta Formation is still under debate, a recent chronostratigraphic synthesis [30] placed deposition of the '*Natica* horizon' at ~50 Ma (Ypresian, early Eocene), because it is bracketed by beds that have been dated via Strontium data to 49.4 Ma and 50.8 Ma, respectively [11,26]. This age assignment agrees very well with biostratigraphic assessments based on correlation of mammal assemblages [31].

Methods

Because the host deposit is only poorly consolidated, sediment samples could be dry-sieved immediately on site over 2 cm mesh screens in order to remove larger pebbles, shells, and shell fragments. The collected residues were then dry-sieved again in the laboratory over >4.0, >2.0, and >0.5 mm mesh screens. Cocoon fossils were picked from the residues of the 2.0–4.0-mm-size fraction under a stereomicroscope using an entomology forceps.

Selected specimens were mounted on stubs, sputter-coated with gold, and examined using a Hitachi S-4300 field emission scanning electron microscope at the Swedish Museum of Natural History. In addition, two cocoon fragments were analysed using Synchrotron-radiation-based X-ray tomographic microscopy (SRXTM) at the Tomography Station of the Materials Science Beamline (TOMCAT) at the Swiss Light Source, Paul Scherrer Institute (Villigen, Switzerland) [32,33]. The specimens were analysed using a beam energy of 10 keV and a 20× objective, resulting in voxel dimensions of 0.325 µm. Projections were acquired over 1501 stepwise increments through a rotation of 180°, and processed and arranged following the methods detailed in [34]. Tomographic reconstructions were made using Avizo®. All material is housed in the palaeobiology collections of the Swedish Museum of Natural History (Stockholm, Sweden), under accession numbers NRM-S089727–089730.

Additional references

26 Reguero M, Goin F, Hospitaleche CA, Dutra T, Marenssi S. 2012 Late Cretaceous/Paleogene West Antarctica terrestrial biota and its intercontinental affinities. *Spring. Briefs Earth Syst. Sci.*, 1–120. (doi:10.1007/978-94-007-5491-1_1)

27 Marenssi SA, Santillana SN, Rinaldi CA. 1998 Stratigraphy of the La Meseta Formation (Eocene), Marambio (Seymour) Island, Antarctica. *Asoc. Paleont. Argent. Publ. Espec.* **5**, 137–146.

28 Iglesias A, Reguero M. 2012 Semillas de angiospermas acuáticas del Eoceno Temprano de Isla Seymour (=Marambio), Península Antártica. In *Abriendo Ventanas al Pasado: Libro de Resúmenes III Simposio-Palaeontología en Chile* (ed M Leppe, JV Aravena, R Villa-Martínez), pp. 72–75. Punta Arenas, Chile.

29 Francis JE et al. 2009 From Greenhouse to Icehouse – the Eocene/Oligocene in Antarctica. In *Antarctic Climate Evolution* (ed. F Florindo, M Siegert), *Developm Earth Environm Sci* **8**, 309–368.

30 Montes M, Nozal F, Santillana SN, Marenssi SA, Olivero E 2013. Mapa Geológico de la Isla Marambio (Seymour). Escala 1:20.000. Primera Edición. Serie Cartográfica Geocientífica Antártica IGME-IAA. Madrid: Instituto Geológico y Minero de España; Buenos Aires: Instituto Antártico Argentino.

31 Gelfo JN, Reguero MA, López GM, Carlini AA, Ciancio MR, Chornogubsky L, Bond M, Goin FJ, Tejedor M 2009. Eocene mammals and continental strata from Patagonia and Antarctic Peninsula. In *Papers on Geology, Vertebrate Paleontology, and Biostratigraphy in Honor of Michael O. Woodburne* (ed. LB Albright III), *Mus. North Arizona Bull.* **64**, 567–592.

32 Stampanoni M, Groso A, Isenegger A, Mikuljan G, Chen Q, Bertrand A, Henein S, Betemps R, Frommherz U, Bohler P, Meister D, Lange M, Abela R. 2006 Trends in

synchrotron-based tomographic imaging: the SLS experience. In *Developments in X-Ray Tomography V* (ed U Bonse), 63180M-14. (doi: 10.1117/12.679497)

33 Friis EM, Marone F, Pedersen KR, Crane PR, Stampanoni M. 2014 Three-dimensional visualization of fossil flowers, fruits, seeds, and other plant remains using Synchrotron radiation X-ray tomographic microscopy (SRXTM): new insights into Cretaceous plant diversity. *J. Paleont.* **88**, 684–701.

34 Marone F, Stampanoni M. 2012 Regridding reconstruction algorithm for real-time tomographic imaging. *J. Synchrotron Radiat.* **19**, 1029–1037. (doi:10.1107/S0909049512032864).

Figure S1

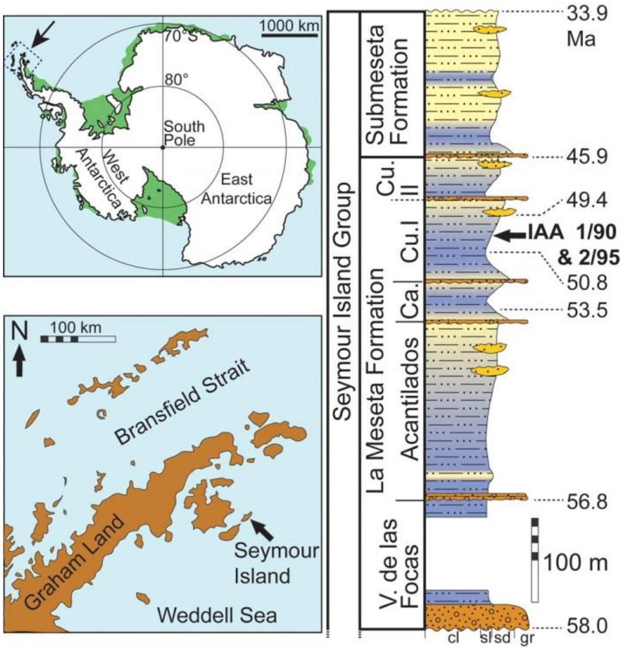

Figure S1. Locality details for the studied fossils: (*a*) map of Antarctica showing the position of the Antarctic Peninsula; (*b*) map of the Antarctic Peninsula showing Seymour Island; (*c*) composite measured section through the La Meseta Formation showing the stratigraphic position of the sampled bed.

Figure S2

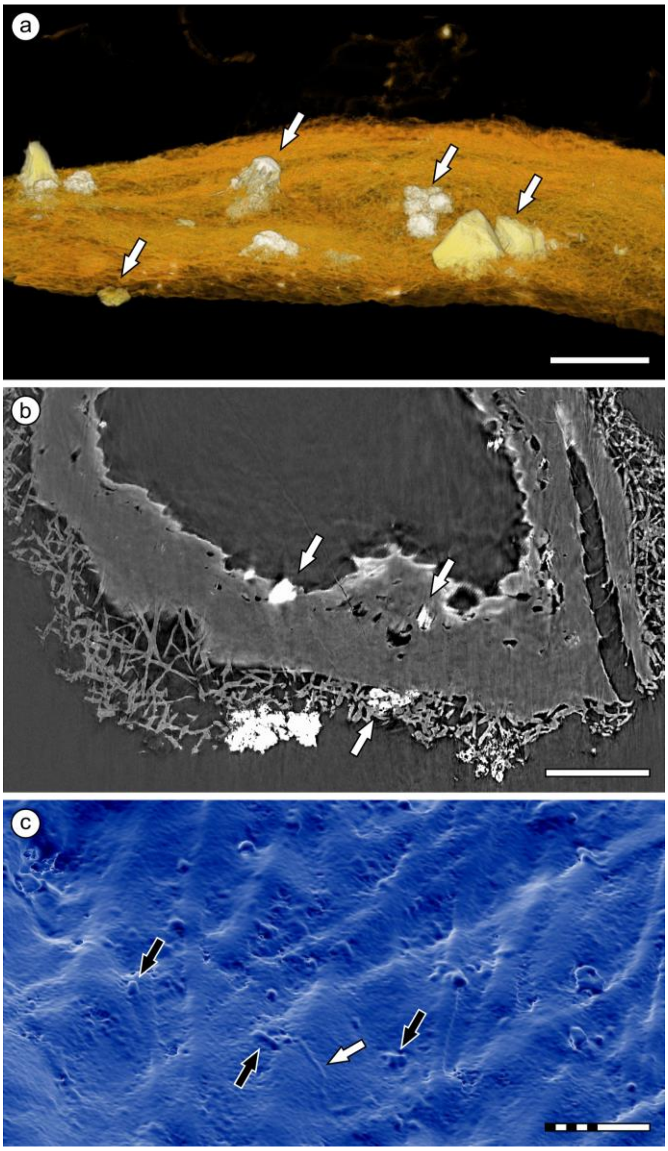

Figure S2. SRXTM reconstructions of an annelid-cocoon wall fragment from the Eocene La Meseta Formation, Seymour/Marambio Island, Antarctica, showing variously sized cocoon-wall inclusions. (*a*) Transparent voltex rendering showing adhering and embedded mineral particles (arrows). (*b*) Orthoslice in oblique tangential section through the clitellate cocoon showing inner solid and outer spongy layer with embedded, highly reflecting mineral particles (arrows). (*c*) Detail of surface rendering of the inner cocoon-wall surface showing embedded structures consistent in overall shape and dimension with an interpretation as included rod-shaped bacilli (black arrows) and a possible spermatozoan fragment (white arrow). Scale bars: (*a*,*b*) = 100 µm; (*c*) = 10 µm.
